# Developmental Regulation of Hepatitis B Virus Biosynthesis by Hepatocyte Nuclear Factor 4α

**DOI:** 10.1371/journal.pone.0005489

**Published:** 2009-05-08

**Authors:** Lie Li, Claudia E. Oropeza, Bruno Sainz, Susan L. Uprichard, Frank J. Gonzalez, Alan McLachlan

**Affiliations:** 1 Department of Microbiology and Immunology College of Medicine, University of Illinois at Chicago, Chicago, Illinois, United States of America; 2 Department of Medicine, College of Medicine, University of Illinois at Chicago, Chicago, Illinois, United States of America; 3 Laboratory of Metabolism, National Cancer Institute, National Institutes of Health, Bethesda, Maryland, United States of America; University of California San Francisco, United States of America

## Abstract

The host cellular factors that promote persistent viral infections *in vivo* are, in general, poorly understood. Utilizing the hepatitis B virus (HBV) transgenic mouse model of chronic infection, we demonstrate that the nuclear receptor, hepatocyte nuclear factor 4α (HNF4α, NR2A1), is essential for viral biosynthesis in the liver. The dependency of HBV transcription on HNF4α links viral biosynthesis and persistence to a developmentally regulated transcription factor essential for host viability.

## Introduction

Persistent viral infections require the coexistence of pathogen and host for extended periods of time without the host resolving the infection or the virus killing the host. Human immunodeficiency virus, herpes simplex viruses, papillomaviruses and hepatitis B and C virus have evolved a variety of strategies to persistently infect man [1]–[4]. HBV chronically infects approximately 400 million people worldwide resulting in about a million deaths per year from liver cirrhosis and hepatocellular carcinoma [5], [6]. Most HBV infections worldwide occur in neonates at or around the time of birth and usually result in persistent infections [6]. Although the immunological immaturity of the neonate is presumed to contribute to these chronic HBV infections, a precise understanding of the molecular events governing HBV persistence during development is lacking.

HBV replicates its genomic DNA by reverse transcription of a pregenomic 3.5kb RNA which is transcribed from covalently closed circular 3.2kb viral genomic DNA in the nucleus of infected hepatocytes [7]. Thus transcriptional regulation plays a central role in controlling viral replication levels [8] and represents a potential antiviral target which has not, to date, been exploited clinically. In cell culture, the binding of hepatocyte nuclear factor 4α (HNF4α) or retinoid X receptor α (RXRα) plus peroxisome proliferator-activated receptor α (PPARα) to the nucleocapsid promoter regulatory elements governs the level of synthesis of this critical HBV pregenomic 3.5kb RNA template [9].

In this study, we demonstrate that the level of HBV transcription and replication throughout early postnatal development correlates with the level of liver HNF4α expression in the HBV transgenic mouse model of chronic HBV infection. The conditional depletion of HNF4α in the liver results in the loss of HBV transcription and replication indicating that this nuclear receptor is a major determinant of viral biosynthesis *in vivo*. These observations indicate that viral transcription, biosynthesis and antigen expression will increase progressively after infection at birth, possibly contributing to persistent infection. Additionally, the essential nature of the HNF4α transcription factor for host viability [10]–[12] may limit the hosts' ability to resolve infection and increase the probability of viral persistence.

## Results

### Characterization of the conditional liver-specific HNF4α deficient HBV transgenic mouse during early postnatal development

Using the HBV transgenic mouse (lineage 1.3.32) model of chronic infection [13], we previously demonstrated that PPARα did not affect the level of viral biosynthesis under normal physiological conditions but did mediate enhanced viral transcription and replication in response to peroxisome proliferators [14]. To determine the role of the transcription factor HNF4α in HBV biosynthesis, we bred HBV transgenic mice with mice carrying a floxed HNF4α (*HNF4α* ^fl/fl^) allele [10] and albumin Cre recombinase (lineage B6.Cg-Tg(Alb-cre)21Mgn/J, Jackson Laboratory) transgene (AlbCre) [15] to generate HBVAlbCreHNF4α^fl/fl^ transgenic mice. The presence of the AlbCre transgene in the HNF4α^fl/fl^ mice results in the postnatal liver-specific loss of exons 4 and 5 of the *HNF4α* gene [10].

**Figure 1:**
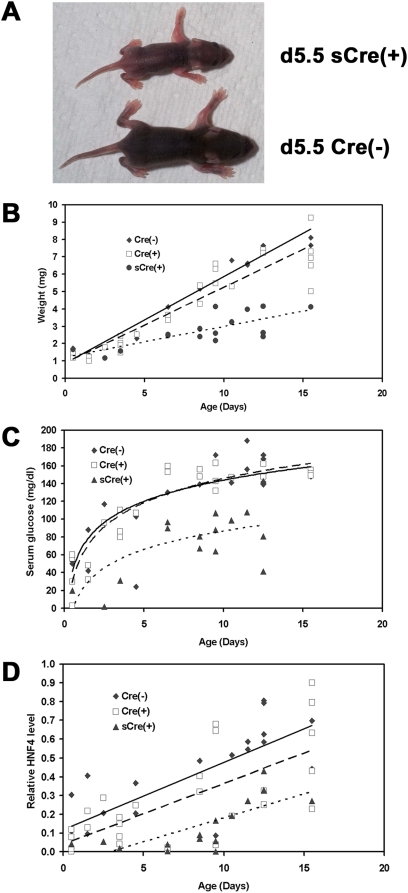
Effects of liver-specific conditional deletion of HNF4α on postnatal growth, serum glucose and HNF4α transcript levels. (A) HBVAlbCre(+)HNF4α^fl/fl^ pup with the small phenotype, sCre(+), and a control HBVAlbCre(−)HNF4α^fl/fl^ littermate, Cre(−), at 5.5 days of age. (B) Effect of postnatal age on body weight. (C) Effect of postnatal age on serum glucose levels. (D) Effect of postnatal age on wild-type HNF4α RNA levels in the liver measured by reverse transcription-quantitative polymerase chain reaction. Cre(−), control HBVAlbCre(−)HNF4α^fl/fl^ mice; Cre(+), HBVAlbCre(+)HNF4α^fl/fl^ mice with approximately wild-type phenotype; sCre(+), HBVAlbCre(+)HNF4α^fl/fl^ mice with the small phenotype.

From the initial crosses generating HBVAlbCreHNF4α^fl/fl^ transgenic mice, it was apparent that some of the pups were not growing at the same rate as their littermates (Figure 1A and B). Indeed, these pups were approximately 50% the weight of their littermates between age 1 and 2 weeks (Figure 1B) and failed to survive past 16 days. Genotyping of these mice demonstrated that all the pups with reduced growth were Cre positive (sCre(+); small, Cre positive). However, many of the Cre positive mice (Cre(+)) within the same litter displayed growth rates closer to normal mice (Cre(−); HNF4α^fl/fl^ transgenic mice lacking the AlbCre transgene) (Figure 1B). While the liver to body weight ratio was similar (3.5+/−0.6%) for the different groups of mice throughout the first two weeks of postnatal development, the sCre(+) mice displayed additional features not observed in the other two groups. sCre(+) mice sera displayed a light yellow-green coloration that became more apparent with age. Additionally, the livers of approximately two thirds of the 1- to 2-week-old sCre(+) mice displayed an olive-yellow coloration rather than the dark red-brown color typically observed in normal mice. Both of these observations are consistent with the sCre(+) mice having an altered phenotype due to loss of HNF4α in the liver. The observed changes in color of the sera and livers may be due to modest increases in circulating bile acids seen in these mice (Cre(−), Cre(+) and sCre(+) were 17+/−9 µmol/l, 21+/−6 µmol/l and 54+/−15 µmol/l, respectively) [10] or an accumulation of the green pigment, biliverdin, a breakdown product of heme [16]. Similar to adult mice lacking HNF4α in their livers, the sCre(+) pups also had reduced circulating glucose (Figure 1C) consistent with lower HNF4α regulated gluconeogenesis in the liver [10], [17].

Wild-type HNF4α transcript levels were measured in the liver using reverse transcription-quantitative polymerase chain reaction analysis specific for HNF4α exons 4 and 5 (Figure 1D) [10]. This analysis demonstrated that while HNF4α levels increased during early postnatal development (Figure 1D), the sCre(+) pups had considerably lower levels of functional HNF4α transcripts compared to the Cre(+) or Cre(−) mice throughout this time period, consistent with their observed phenotype. Additionally, sCre(+) mice with altered sera and liver coloration were never observed after 16 days, suggesting that these pups either died due to the lack of HNF4α in their hepatocytes or partially recovered as synthesis of HNF4α returned to an adequate level during liver development presumably due to the outgrowth of hepatocytes where the HNF4α gene was not efficiently deleted by the Cre recombinase.

### Characterization of viral transcription in the conditional liver-specific HNF4α deficient HBV transgenic mouse during early postnatal development

**Figure 2:**
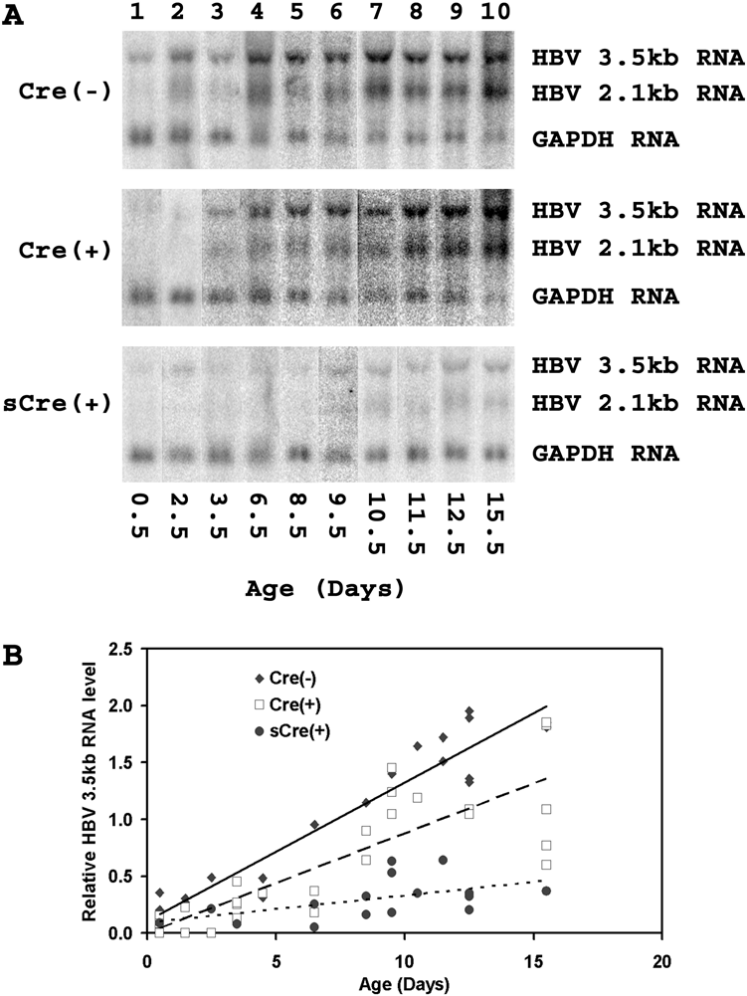
HBV RNA transcripts in the livers of HBV transgenic mice throughout early postnatal development. (A) RNA (Northern) filter hybridization analysis of representative examples of HBV transcripts at various stages of postnatal liver development. The glyceraldehyde 3-phosphate dehydrogenase (GAPDH) transcript was used as an internal control for the quantification of the HBV 3.5 and 2.1kb RNAs. The probes used were HBV*ayw* genomic DNA plus GAPDH cDNA[26]. Cre(−), control HBVAlbCre(−)HNF4α^fl/fl^ mice; Cre(+), HBVAlbCre(+)HNF4α^fl/fl^ mice with approximately wild-type phenotype; sCre(+), HBVAlbCre(+)HNF4α^fl/fl^ mice with the small phenotype. (B) Quantitative analysis of the HBV 3.5kb transcript in HBVAlbCreHNF4α^fl/fl^ transgenic mice. Trend lines were calculated using linear regression analysis.

**Figure 3:**
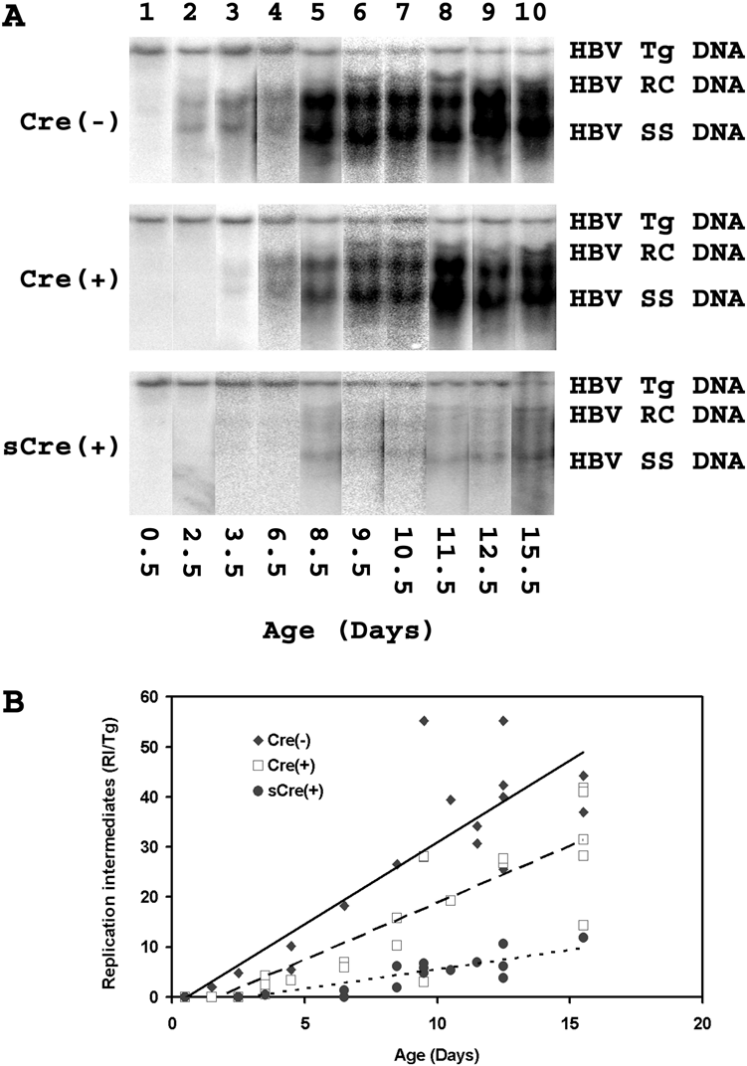
HBV DNA replication intermediates in the livers of HBV transgenic mice throughout early postnatal development. (A) DNA (Southern) filter hybridization analysis of representative examples of HBV replication intermediates (RI) at various stages of postnatal liver development. The HBV transgene (Tg) was used as an internal control for the quantification of the HBV replication intermediates. The probe used was HBV*ayw* genomic DNA[26]. Cre(−), control HBVAlbCre(−)HNF4α^fl/fl^ mice; Cre(+), HBVAlbCre(+)HNF4α^fl/fl^ mice with approximately wild-type phenotype; sCre(+), HBVAlbCre(+)HNF4α^fl/fl^ mice with the small phenotype; Tg , HBV transgene; RC, HBV relaxed circular replication intermediates; SS, HBV single stranded replication intermediates. (B) Quantitative analysis of the HBV replication intermediates in HBVAlbCreHNF4α^fl/fl^ transgenic mice. Trend lines were calculated using linear regression analysis.

These distinct groups of HBV transgenic mice (Cre(−), Cre(+) and sCre(+)) exhibiting different levels of functional HNF4α expression throughout early postnatal development permitted the role of HNF4α in viral transcription and replication in the liver to be determined (Figures 2 and 3). Wild-type HBV transgenic mice (Cre(−)) displayed about a ten-fold increase in HBV 3.5kb RNA from birth to two weeks of age (Figure 2). During the same time period, HNF4α levels increased approximately five-fold (Figure 1D). The Cre(+) mice which display slightly reduced levels of HNF4α in their liver also showed reduced viral transcription (Figure 2) while the sCre(+) mice, exhibiting a major reduction in HNF4α levels throughout this period of development, displayed drastically reduced levels of HBV transcription (Figure 2). Together these data demonstrate a clear correlation between the abundance of HBV transcripts *in vivo* and the level of HNF4α during development indicating that HNF4α is essential for viral RNA synthesis.

### Characterization of viral replication in the conditional liver-specific HNF4α deficient HBV transgenic mouse during early postnatal development

The lower levels of viral 3.5kb pregenomic RNA associated with diminished HNF4α abundance in the liver resulted in similarly reduced HBV replication (Figure 3). In agreement with the observed reduction of HNF4α (Figure 1D), the Cre(+) mice displayed reduced levels of viral DNA synthesis throughout the first two weeks of postnatal development and the appearance of viral replication intermediates was delayed by at least a day compared to the Cre(−) mice (Figure 3). Likewise, the sCre(+) mice displayed a drastic reduction in viral replication consistent with their much lower level of HBV transcripts (Figures 2 and 3) and considerably lower levels of HNF4α expression (Figure 1D). Therefore it appears that under normal physiological conditions a single transcription factor, HNF4α, controls the early developmental expression of HBV transcripts and consequently viral replication *in vivo*.

## Discussion

HNF4α binds the proximal regulatory element of the nucleocapsid promoter and activates the expression of the HBV 3.5kb pregenomic RNA in cell culture [9]. Likewise, the analysis of HBV transcription and replication throughout early postnatal development in wild-type HBV transgenic mice and mice conditionally deleted for HNF4α suggest that it serves the same function *in vivo*. HNF4α levels increase in the liver throughout the first two weeks of postnatal development and is associated with a concomitant increase in both HBV RNA transcripts and DNA replication intermediates. The liver-specific conditional disruption of HNF4α during this period of development was associated with two distinct phenotypes. Mice with a severe HNF4α deficiency in the liver displayed reduced growth, altered glucose homeostasis and a postnatal lethal phenotype. These mice synthesized limited amounts of HBV transcripts and replication intermediates. Mice that were close to displaying a normal phenotype with respect to growth and glucose homeostasis exhibited a modest decrease in liver HNF4α levels that was associated with a similar decrease in both viral RNA and DNA synthesis. These observations indicate that HBV transcription and hence viral replication is dependent on HNF4α. Although it is likely that HNF4α directly activates HBV transcription [9], it is also possible that HNF4α might control the expression of additional factors necessary for efficient viral transcription and replication *in vivo*. Additionally, the hepatotoxicity resulting from extensive HNF4α loss may contribute to the observed reduction in HBV transcription and replication by unknown mechanisms.

These studies highlight an interesting relationship between HBV biosynthesis and liver-specific gene expression. We show HNF4α is essential *in vivo* to sustain active viral transcription and subsequent replication. Similarly, HNF4α is required for HBV host viability. The absence of HNF4α or its conditional loss from the liver is fatal in the mouse [10]–[12]. Haploinsufficiency of HNF4α in man is responsible for maturity onset diabetes of the young type 1 (MODY1) [18], [19]. Humans with a complete loss of HNF4α have not been reported presumably because, as in mice, it results in early embryonic lethality. Curiously, the adaptation of HBV to utilize HNF4α, an essential host gene product, guarantees constitutive replication of its genome as long as the host is viable. This adaptation may contribute to HBV persistence and offer the virus a significant selective advantage over other pathogens that utilize cellular components that are less important to host survival.

The vast majority of HBV infections acquired in adolescence and early adulthood are successfully cleared by the host. In contrast, most HBV infections that occur at birth result in chronic infection [20] despite the ability of neonates to mount an antiviral immune response if vaccinated [21]. The parallel increase in HNF4α expression and HBV biosynthesis levels observed during postnatal development in our study suggest that neonatal HBV infection is associated with a slow developmentally regulated increase in viral transcription, replication and antigen expression which may contribute to life long chronic infection [20], [22]. Therapeutic interventions that alter the expression levels of viral gene products at this stage of development, such as the fibrate class of hyperlipidemic drugs [14], might modulate the immune response of the host to viral antigens and help immunological resolution of these neonatal infections.

The observation that HBV is critically dependent on at least one cellular factor offers a unique target for antiviral therapy. However this opportunity comes with the difficult challenge of inhibiting HNF4α without completely eliminating its essential cellular functions. Nevertheless, mice exhibiting only a modest decrease in HNF4α levels displayed a detectable reduction in HBV RNA and DNA levels while maintaining virtually normal growth and glucose homeostasis (Figure 1). Furthermore, newborn pups express enough HNF4α for viability but not enough to support HBV replication. If HNF4α activity levels can be attenuated clinically to limit HBV biosynthesis while minimally affecting liver physiology, this might offer a novel therapeutic strategy to complement currently available antiviral drugs targeting the HBV reverse transcriptase [20]. Metformin, a drug widely used to treat type 2 diabetes, reduces peroxisome proliferator-activated receptor γ coactivator 1α expression and gluconeogenesis in the liver suggesting pharmacological intervention may be successful in decreasing HNF4α-dependent HBV transcription and replication [23], [24].

## Materials and Methods

### Ethics statement

All animal experiments were performed according to institutional guidelines with Institutional Biosafety and Animal Care Committee approval.

### Transgenic mice

We bred HBV transgenic mice (lineage 1.3.32) [13] with mice carrying a floxed HNF4α (*HNF4α* ^fl/fl^) allele [10] and albumin Cre (lineage B6.Cg-Tg(Alb-cre)21Mgn/J, Jackson Laboratory) transgene (AlbCre) [15] to generate HBVAlbCreHNF4α^fl/fl^ transgenic mice. All the mouse strains had been crossed for twelve generations onto the SV129 genetic background [25] prior to generating the HBVAlbCreHNF4α^fl/fl^ transgenic mice. Mice were screened for the HBV transgene, the AlbCre transgene and the floxed HNF4α allele by polymerase chain reaction (PCR) analysis of tail DNA [26]. The HBV transgene was identified by PCR analysis using the oligonucleotides, 5′-TCGATACCTGAACCTTTACCCCGTTGCCCG-3′ (oligo XpHNF4-1, HBV coordinates 1133 to 1159) and 5′-TCGAATTGCTGAGAGTCCAAGAGTCCTCTT-3′ (oligo CpHNF4-2, HBV coordinates 1683 to 1658), and 1 µl of tail DNA. A PCR product of 551 base pairs indicated the presence of the HBV transgene. The AlbCre transgene was identified by PCR analysis using the oligonucleotides, 5′-CCAGCTAAACATGCTTCATCGTCG-3′ (oligo CRE-1) and 5′-ATTCTCCCACCGTCAGTACGTGAG-3′ (oligo CRE-2), and 1 µl of tail DNA. A PCR product of 300 base pairs indicated the presence of the Cre transgene. The HNF4α wild-type and floxed alleles were identified by PCR analysis using the oligonucleotides, 5′-AGAATGACCCTGAAGCACCAGG-3′ (oligo prH4GTLP1-F1) and 5′-GCCAGAGGTCTGTGAAACAAGG-3′ (oligo prH4GT-LP1-R1), and 1 µl of tail DNA. PCR products of 180 and 241 base pairs indicated the presence of the wild-type and floxed HNF4α alleles, respectively [10]. Serum glucose levels were measured using a SureStep blood glucose meter (Lifescan).

### HBV DNA and RNA analysis

Total DNA and RNA were isolated from liver of HBV transgenic mice as described [27], [28]. DNA (Southern) filter hybridization analyses were performed using 20 µg of *Hin*dIII digested DNA [27]. Filters were probed with ^32^P-labeled HBV*ayw* genomic DNA [29] to detect HBV sequences. RNA (Northern) filter hybridization analyses were performed using 10 µg of total cellular RNA as described [27]. Filters were probed with ^32^P-labeled HBV*ayw* genomic DNA to detect HBV sequences and mouse glyceraldehyde 3-phosphate dehydrogenase (GAPDH) cDNA to detect the GAPDH transcript used as an internal control [30]. Filter hybridization analyses were quantitated by phosphorimaging using a Packard Cyclone Storage Phosphor System.

Reverse transcription-quantitative polymerase chain reaction was used to measure the level of HNF4α transcripts in mouse liver RNA. After DNase I treatment, 1 µg of RNA was used for cDNA synthesis using the TaqMan reverse transcription reagents (Applied Biosystems, Foster City, CA), followed by real-time PCR quantification using SYBR Green and an Applied Biosystems 7300 real-time thermocycler (Applied Biosystems). Thermal cycling consisted of an initial denaturation step for 10 min at 95°C followed by 40 cycles of denaturation (15 sec at 95°C) and annealing/extension (1 min at 60°C). The relative HNF4α RNA expression levels were estimated using the ΔΔCt method with normalization to mouse GAPDH RNA. The PCR primers used were 5′-TCAACGCGCTCCTGC-3′ (HNF4α exon 4 sense primer), 5′-AATCTTCTTTGCCCGAATGT-3′ (HNF4α exon 5 antisense primer), 5′-TCTGGAAAGCTGTGGCGTG-3′ (mouse GAPDH sense primer) and 5′-CCAGTGAGCTTCCCGTTCAG-3′ (mouse GAPDH antisense primer).

## References

[1] Geeraert L, Kraus G, Pomerantz RJ (2008). Hide-and-seek: the challenge of viral persistence in HIV-1 Infection.. Annu Rev Med.

[2] Knipe DM, Cliffe A (2008). Chromatin control of herpes simplex virus lytic and latent infection.. Nat Rev Micro.

[3] Stanley M (2008). Immunobiology of HPV and HPV vaccines.. Gynecol Oncol.

[4] Gale M, Foy EM (2005). Evasion of intracellular host defence by hepatitis C virus.. Nature.

[5] Lok AS, Heathcote EJ, Hoofnagle JH (2001). Management of hepatitis B: 2000 - Summary of a workshop.. Gastroenterology.

[6] Lavanchy D (2004). Hepatitis B virus epidemiology, disease burden, treatment, and current and emerging prevention and control measures.. J Viral Hepat.

[7] Will H, Reiser W, Weimer T, Pfaff E, Buscher M (1987). Replication strategy of human hepatitis B virus.. J Virol.

[8] Tang H, Banks KE, Anderson AL, McLachlan A (2001). Hepatitis B virus transcription and replication.. Drug News Perspect.

[9] Tang H, McLachlan A (2001). Transcriptional regulation of hepatitis B virus by nuclear hormone receptors is a critical determinant of viral tropism.. Proc Natl Acad Sci USA.

[10] Hayhurst GP, Lee YH, Lambert G, Ward JM, Gonzalez FJ (2001). Hepatocyte nuclear factor 4α (nuclear receptor 2A1) is essential for maintenance of hepatic gene expression and lipid homeostasis.. Mol Cell Biol.

[11] Parviz F, Matullo C, Garrison WD, Savatski L, Adamson JW (2003). Hepatocyte nuclear factor 4α controls the development of a hepatic epithelium and liver morphogenesis.. Nature Genet.

[12] Chen WS, Manova K, Weinstein DC, Duncan SA, Plump AS (1994). Disruption of the HNF-4 gene, expressed in visceral endoderm, leads to cell death in embryonic ectoderm and impaired gastrulation of mouse embryos.. Genes Dev.

[13] Guidotti LG, Matzke B, Schaller H, Chisari FV (1995). High-level hepatitis B virus replication in transgenic mice.. J Virol.

[14] Guidotti LG, Eggers CM, Raney AK, Chi SY, Peters JM (1999). *In vivo* regulation of hepatitis B virus replication by peroxisome proliferators.. J Virol.

[15] Postic C, Shiota M, Niswender KD, Jetton TL, Chen Y (1999). Dual roles for glucokinase in glucose homeostasis as determined by liver and pancreatic beta cell-specific gene knock-outs using Cre recombinase.. J Biol Chem.

[16] Fenech FF, Bannister WH, Grech JL (1967). Hepatitis with biliverdinaemia in association with indomethacin therapy.. Br Med J.

[17] Rhee J, Inoue Y, Yoon JC, Puigserver P, Fan ML (2003). Regulation of hepatic fasting response by PPARγ coactivator-1α (PGC-1): Requirement for hepatocyte nuclear factor 4α in gluconeogenesis.. Proc Natl Acad Sci USA.

[18] Yamagata K, Furuta H, Oda N, Kaisaki PJ, Menzel S (1996). Mutations in the hepatocyte nuclear factor-4α gene in maturity- onset diabetes of the young (MODY1).. Nature.

[19] Shih DQ, Dansky HM, Fleisher M, Assmann G, Fajans SS (2000). Genotype/phenotype relationships in HNF-4α/MODY1 - Haploinsufficiency is associated with reduced apolipoprotein(AII), apolipoprotein(CIII), lipoprotein(a), and triglyceride levels.. Diabetes.

[20] Dienstag JL (2008). Hepatitis B virus infection.. N Engl J Med.

[21] Del Canho R, Grosheide PM, Voogd-Schotanus M, Huisman WM, Heijtink RA (1994). Immunogenicity of two different dosages (10 and 5 µg) of recombinant DNA hepatitis B vaccine in healthy neonates.. Vaccine.

[22] Milich DR, Jones JE, Hughes JL, Price J, Raney AK (1990). Is a function of the secreted hepatitis B e antigen to induce immunologic tolerance *in utero*?. Proc Natl Acad Sci USA.

[23] Shaw RJ, Lamia KA, Vasquez D, Koo SH, Bardeesy N (2005). The kinase LKB1 mediates glucose homeostasis in liver and therapeutic effects of metformin.. Science (New York), N Y.

[24] Shlomai A, Paran N, Shaul Y (2006). PGC-1α controls hepatitis B virus through nutritional signals.. Proc Natl Acad Sci USA.

[25] Lee SST, Pineau T, Drago J, Lee EJ, Owens JW (1995). Targeted disruption of the α isoform of the peroxisome proliferator-activated receptor gene in mice results in abolishment of the pleiotropic effects of peroxisome proliferators.. Mol Cell Biol.

[26] Anderson AL, Banks KE, Pontoglio M, Yaniv M, McLachlan A (2005). Alpha/beta interferon differentially modulates the clearance of cytoplasmic encapsidated replication intermediates and nuclear covalently closed circular hepatitis B virus (HBV) DNA from the livers of hepatocyte nuclear factor 1α-null HBV transgenic mice.. J Virol.

[27] Sambrook J, Fritsch EF, Maniatis T (1989). Molecular cloning: a laboratory manual..

[28] Chomczynski P, Sacchi N (1987). Single-step method of RNA isolation by acid guanidinium thiocyanate-phenol-chloroform extraction.. Anal Biochem.

[29] Galibert F, Mandart E, Fitoussi F, Tiollais P, Charnay P (1979). Nucleotide sequence of the hepatitis B virus genome (subtype ayw) cloned in *E. coli*.. Nature.

[30] Sabath DE, Broome HE, Prystowsky MB (1990). Glyceraldehyde-3-phosphate dehydrogenase mRNA is a major interleukin 2- induced transcript in a cloned T-helper lymphocyte.. Gene.

